# Structural insight into _D_-xylose utilization by xylose reductase from *Scheffersomyces stipitis*

**DOI:** 10.1038/s41598-018-35703-x

**Published:** 2018-11-28

**Authors:** Hyeoncheol Francis Son, Sun-Mi Lee, Kyung-Jin Kim

**Affiliations:** 1 School of Life Sciences, KNU Creative BioResearch Group, Kyungpook National University, Daegu, 41566 Republic of Korea; 2 KNU Institute for Microorganisms, Kyungpook National University, Daegu, 41566 Republic of Korea; 3 Clean Energy Research Center, Korea Institute of Science and Technology (KIST), Seoul, 02792 Republic of Korea

## Abstract

Lignocellulosic biomass, of which _D_-xylose accounts for approximately 35% of the total sugar, has attracted attention as a future energy source for biofuel. To elucidate molecular mechanism of _D_-xylose utilization, we determined the crystal structure of _D_-xylose reductase from *Schefferzomyces stipitis* (*Ss*XR) at a 1.95 Å resolution. We also determined the *Ss*XR structure in complex with the NADPH cofactor and revealed that the protein undergoes an open/closed conformation change upon NADPH binding. The substrate binding pocket of *Ss*XR is somewhat hydrophobic, which seems to result in low binding affinity to the substrate. Phylogenetic tree analysis showed that AKR enzymes annotated with bacterial/archaeal XRs belonged to uncharacterized AKR families and might have no XR function, and yeast/fungi derived enzymes, which belong to the same group with *Ss*XR, can be candidates for XR to increase xylose consumption.

## Introduction

Industrialization and population growth lead to increased energy demands, and interest in alternative energy has increased because of fossil fuel depletion and environmental problems. Lignocellulosic biomass has attracted attention as a future energy source for biofuel and polymer production, because it is abundant, sustainable and does not compete with edible resources^1–3^. Particularly, the recent application of lignocellulosic biomass has expanded not only to conventional bioethanol but also to biodiesel production, and thus numerous studies have been conducted to solve the energy problem using ligonocellulosic biomass^4^. However, biofuel producing organisms, such as *Saccharomyces cerevisiae* and *Yarrowia lipolytica*, lack the pentose metabolic pathway, which limits the use of lignocellulosic biomass as a resource^4,5^. Therefore, many studies have been conducted to overcome the limitation of pentose utilization by introducing the pentose metabolic pathway from heterologous organisms^5–11^.

**Figure 1:**
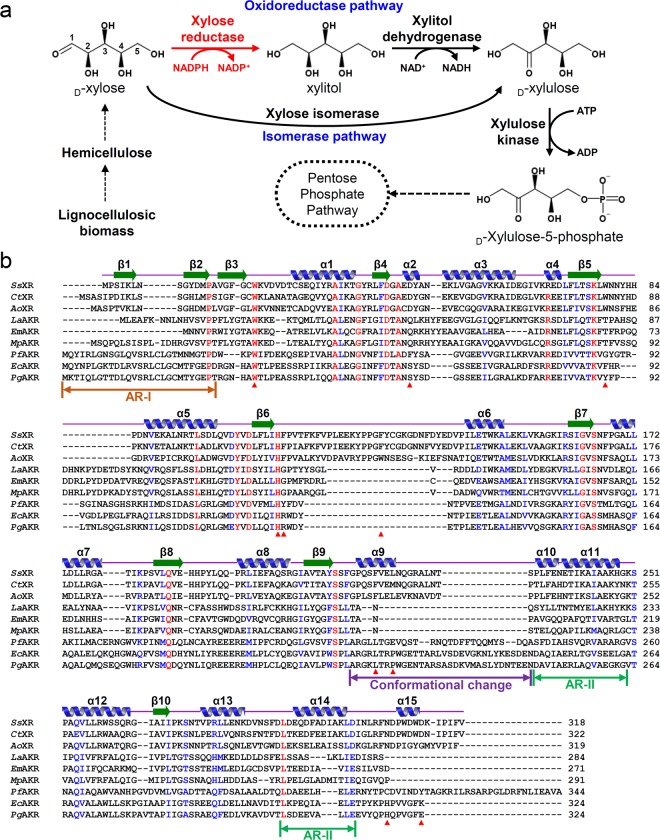
_D_-Xylose metabolic pathway and amino acid sequence alignment of XRs. (**a**) _D_-xylose metabolic pathway. (**b**) Amino acid sequence alignment of XRs, UNC AKR I, and UNC AKR II. The secondary structure elements are drawn based on the structure of *Ss*XR in the closed conformation. The residues involved in the formation of _D_-xylose substrate binding pocket of *Ss*XR are indicated by red colored triangles. *Ss*XR, *Ct*XR, and *Ao*XR are abbreviation of XRs from *Scheffersomyces stipitis*, *Candida tenuis*, and *Aspergillus oryzae*, respectively. *Pf*AKR, *Ec*AKR, *Pa*AKR, *La*AKR, *Em*AKR, *Mp*AKR are abbreviation of AKR from *Legionella anisa*, *Endozoicomonas montiporae*, *Methylovulum psychrotolerans*, *Pseudomonas fluorescens*, *Escherichia coli*, and *Pantoea agglomerans*, respectively.

_D_-Xylose, which accounts for approximately 35% of the total sugar in lignocellulosic biomass, is considered a good industrial resource^12–15^. _D_-Xylose can be converted to _D_-xylulose by two different biological pathways: the oxidoreductase pathway and isomerase pathway (Fig. 1a). In the isomerase pathway, xylose isomerase (XI) directly converts _D_-xylose to _D_-xylulose, and has a high theoretical yield because XI enzyme does not require any cofactors. Therefore, various heterologous XI enzymes have been introduced into *S*. *cerevisiae* and *Y*. *lipolytica* to assimilate _D_-xylose and recently, the crystal structures of XI from *Piromyces* sp. (*Ps*XI) were determined^4,16–22^. In the oxidoreductase pathway, two enzymes, xylose reductase (XR) and xylitol dehydrogenase (XDH), are used to convert _D_-xylose to _D_-xylulose using xylitol as an intermediate (Fig. 1a), and XR/XDH enzymes derived from *Scherrsomyces stipitis* (*Ss*XR, *Ss*XDH, respectively) have been introduced into *S*. *cerevisiae* and *Y*. *lipolytica* for _D_-xylose intake^4,23,24^. XR/XDH enzymes can offer higher metabolic fluxes than the XI enzymes, however _D_-xylose intake by the oxidoreductase pathway causes cofactor imbalance, because XR and XDH use NADPH and NAD^+^ as cofactors, respectively (Fig. 1a). Although several protein engineering trials to change the cofactor specificity were performed^25–28^, there has been not fundamental solution for cofactor imbalance problem, due to the lack of the crystal structure of *Ss*XR and *Ss*XDH.

Aldo-keto reductases (AKRs), which include _D_-xylose reductase, are found in almost all organisms, and has broad substrate specificity for sugars, lipid aldehydes, ketosteroids, ketoprostaglandins, and chemical carcinogens as substrates^29–34^. Based on amino acid sequence similarities, more than 190 AKR members are divided into 16 families, with XR enzymes belonging to the AKR family 2B^35^. Until now, only one XR structure derived from *Candida tenuis* (*Ct*XR) was reported^36^, however, the crystal structure of *Ss*XR used in _D_-xylose consumption by introduction into *S*. *cerevisiae* and *Y*. *lipolytica* has not been determined yet. Here, we report the crystal structures of _D_-xylose reductase from *S*. *stipitis* (*Ss*XR) in its apo form and in complex with NADPH cofactor. We revealed that the protein undergoes an open/closed conformation change upon the binding of NADPH and has somewhat hydrophobic _D_-xylose binding pocket. Phylogenetic tree analysis showed that bacteria/archaea derived XR-annotated AKRs might have no XR activity, and yeast/fungi derived XRs can be candidate to increase xylose consumption.

## Results and Discussion

### Overall structure of *Ss*XR

**Figure 2:**
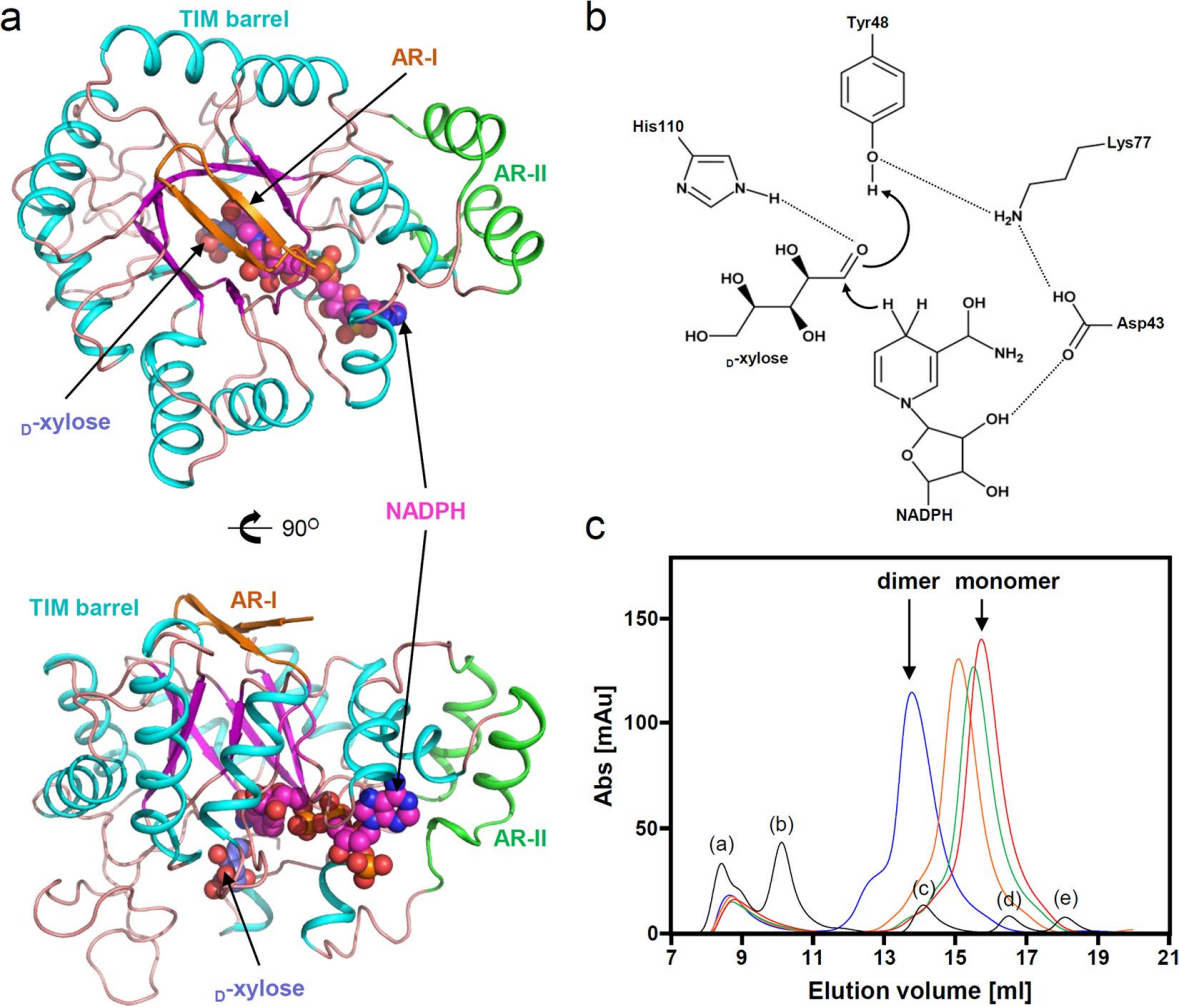
Overall structure of *Ss*XR. (**a**) The monomeric structure of *Ss*XR. The monomeric structure of *Ss*XR is presented as a cartoon diagram. α-helices, β-strands, and loops in core domain are distinguished cyan, magenta, and orange colors, respectively, and auxiliary region I, and II (AR-I and AR-II) are distinguished with orange, and green colors, respectively, and labeled. The bound NADPH cofactors and modeled _D_-xylose substrates are presented as sphere models with magenta and light blue colors, respectively. The bottom figure is rotated by 90 degree horizontally from the top figure. (**b**) Putative catalytic mechanism of *Ss*XR. Dashed likes show hydrogen bonds. (**c**) Size-exclusion chromatography of *Ss*XR. The *Ss*XR samples in condition of 0, 50, 100, and 150 mM NaCl are distinguished with blue, orange, green, and red colors, respectively. The *Ss*XR with 0 and 150 mM NaCl are eluted as a dimeric and monomeric form, respectively. The standard graph was drawn with black color. (**a**) Indicates void volume and (**b**–**d**), € indicate the standard samples of ferritin (440 kDa), conalbumin (75 kDa), carbonic anhydratse (29 kDa), and ribonuclease A (13.7 kDa), respectively.

To elucidate the molecular mechanism of *Ss*XR, we determined its crystal structure at 1.95 Å resolution. The refined structure was in good agreement with the X-ray crystallographic statistics for bond angles, bond lengths, and other geometric parameters (Table 1). The overall structure of *Ss*XR is similar to that of the AKR superfamily enzymes. The monomeric structure of *Ss*XR is composed of 15 α-helices (α1-α15) and 10 β-strands (β1-β10) (Fig. 1b). The monomeric structure of *Ss*XR consists of a core domain and two auxiliary regions (ARs), AR-I and AR-II. The core domain consists of 13 α-helices (α1-α10, α12-α13, and α15) and eight β-strands (β3-β10) and forms a TIM-barrel motif (Figs 1b and 2a). As the conventional TIM-barrel motif, in *Ss*XR eight parallel β-strands (β3-β10) are arranged in a cylindrical shape with eight surrounding α-helices (α1, α3, α5-α8, and α12-α13). Four α-helices (α2, α9-α10, and α15) are located at the back of the TIM-barrel motif and contribute to binding of the NADPH cofactor (Fig. 2a). AR-I is composed of two β-strands (β1-β2) and is located on the opposite side of the TIM-barrel. AR-II consists of two α-helices (α11 and α14) and is positioned next to the α12 helix (Fig. 2a). As reported in other enzymes belonging to AKR families, four catalytic residues, Asp43, Tyr48, Lys77, and His110, are also conserved in the *Ss*XR, and catalytic mechanism can be proposed^37^ (Fig. 2b).

**Table 1:** Data collection and refinement statistics.

	*Ss*XR_Apo	*Ss*XR_NADPH
**Data collection**		
Space group	P2_1_2_1_2_1_	P4_2_2_1_2
Cell dimensions		
*a*, *b*, *c* (Å)	69.2,87.2,122.6	97.7,97.7,160.1
α, β, γ (°)	90.00, 90.00, 90.00	90.00, 90.00, 90.00
Resolution (Å)	50.00-1.95 (1.98–1.95)	50.00–2.00 (2.03–2.00)
*R*_sym_ or *R*_merge_ (%)	9.7 (20.8)	11.1 (27.3)
*R*_meas_ (%)	10.6(23.0)	11.6 (29.8)
*I*/σ (*I*)	55.2 (15.1)	26.3 (3.3)
Completeness (%)	98.8 (99.8)	98.6 (96.6)
Redundancy	6.7 (5.4)	8.7 (5.4)
**Refinement**		
Resolution (Å)	50.00–1.95	50.00–2.00
No. reflections	51177	49336
*R*_work_/*R*_free_	18.9 (23.6)	17.7 (22.4)
No. atoms	5491	5526
Protein	5084	5068
Ligand/ion	12	96
Water	395	362
*B*-factors (Å^2^)	36.514	29.337
Protein	36.152	28.988
Ligand/ion	37.886	38.841
Water	42.366	34.135
R.m.s. deviations		
Bond lengths (Å)	0.0184	0.0189
Bond angles (°)	1.7601	1.983
Ramachandran statistics		
Favoured (%)	98	96
Allowed (%)	2	4
Outliers (%)	0	0

It has been reported that AKR enzymes function as a monomer, dimer, and tetramer^37^ and *Ss*XR is known to exist as a dimer in the presence of 50 mM NaCl^38^. In our current structure, there are two *Ss*XR polypeptide chains in an asymmetric unit forming a dimer. PISA and EPPIC calculations also showed that the protein exists as a dimer, although the interaction was quite low. We then performed size-exclusion chromatography experiments under various NaCl concentrations. Unexpectedly, *Ss*XR generally separated into monomers in 50 and 100 mM NaCl and completely separated into monomers in150 mM NaCl, although it existed as dimers in the absence of NaCl (Figs 2c, S1). Based on these results, we suggest that *Ss*XR exists as a monomer under the physiological NaCl concentration and tends to form a dimer in the presence of low NaCl concentrations. Furthermore, in order to compare the enzyme activity according to the oligomec state, kinetic values at 0 mM and 150 mM were measured. Interestingly, all values at 0 mM NaCl condition were better than 150 mM NaCl, indicating that oligomer formation affects enzyme activity.

### Open/closed conformation for cofactor binding of *Ss*XR

**Figure 3:**
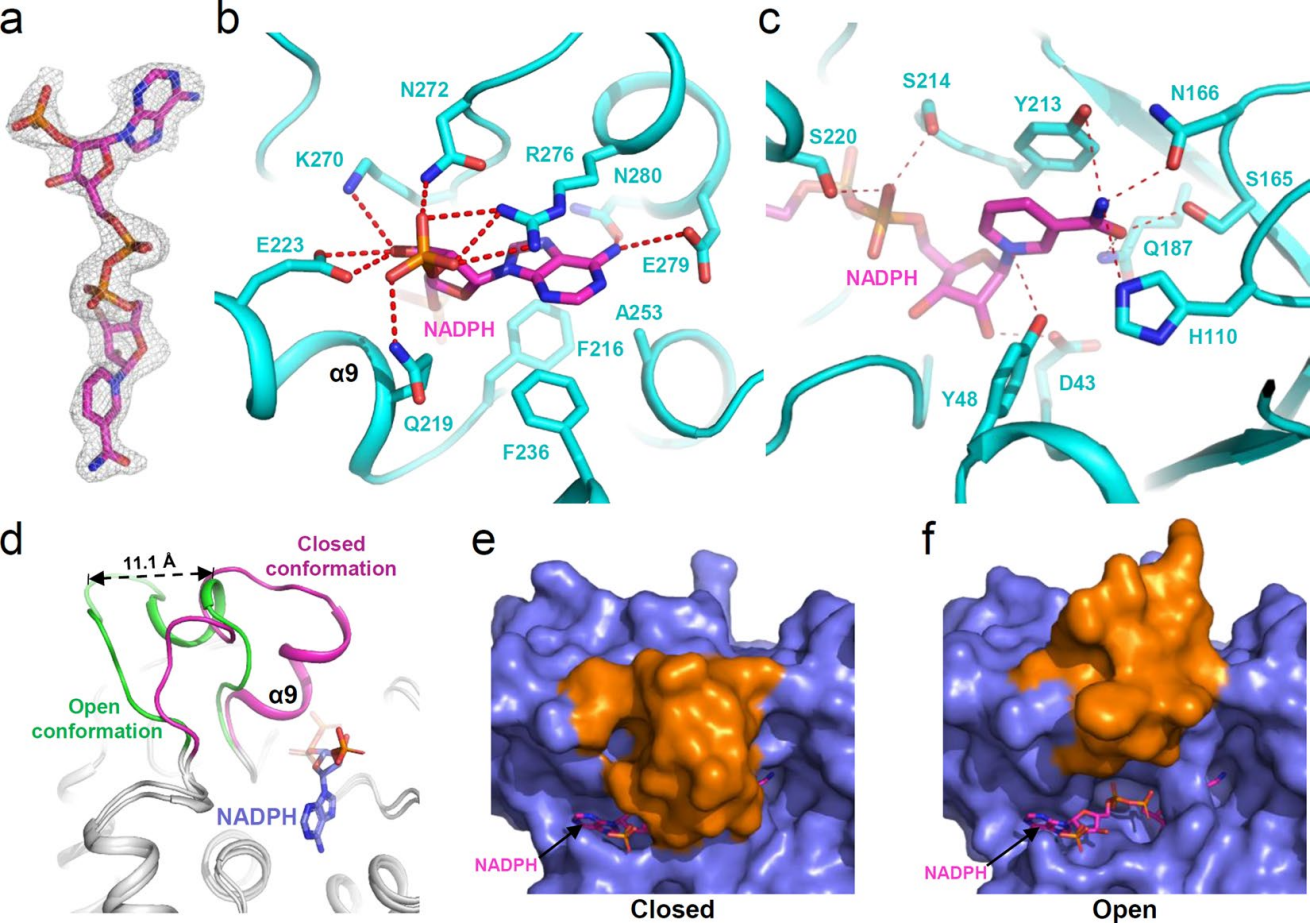
NADPH cofactor binding mode of *Ss*XR. (**a**) Electron density map of the bound NADPH cofactor. The Fo-Fc electron density map of NADPH cofactor is shown with a gray-colored mesh, and contour 2.5 σ. The NADPH cofactor is shown as a stick model with magenta color. (b,c) NADPH cofactor binding mode of *Ss*XR. The binding mode of nucleotide part (**b**), and pyrophosphate, ribose ring, and nicotinamide ring (**c**) of *Ss*XR. The structure of *Ss*XR is shown as a cartoon diagram with cyan color. The residues involved in the NADPH binding are shown as stick models and labeled appropriately. The bound NADPH cofactor is shown as a stick model with a magenta color. Red color dotted lines indicate hydrogen bonds contributing to NADPH binding. (**d**) Structural comparison of the open and closed conformation of *Ss*XR. The TIM-barrel motif of the open and closed conformation of *Ss*XR were superposed, and the relative positions of the α9 were compared. The moving part of the open and closed conformation of *Ss*XR were with green and magenta colors, respectively, and labeled. The bound NADPH cofactor was shown as sphere model with light-blue color. (**e**,**f**) The open and closed conformation of *Ss*XR. The closed (**e**) and open (**f**) conformations of *Ss*XR were shown as surface model. The moving part and rigid body were distinguished with orange and light-blue colors, respectively. The bound NADPH cofactors were shown as stick model with magenta color.

AKR superfamily enzymes utilize NADH and/or NADPH as a cofactor. To examine the cofactor preference of *Ss*XR, we performed kinetic analysis using NADH and NADPH as cofactors. At the physiological condition, the K_m_ values for NADPH and NADH were 0.0277 and 0.136 mM, respectively, and the *k*_cat_ values for NADPH and NADH were 7.63 and 2.39s^−1^, respectively (Table 2). These results indicate that *Ss*XR prefers NADPH as a cofactor and has a 15-fold higher *k*_cat_/K_m_ value for NADPH than for NADH. To elucidate the cofactor binding mode of *Ss*XR, we determined the crystal structure of *Ss*XR in complex with NADPH at 2.0 Å resolution (Table 1, Fig. 3a). The NADPH cofactor bound at the back of the TIM barrel motif (Fig. 2a). The residues Phe216, Gln219, Glu223, Phe236, Ala253, Lys270, Asn272, Arg276, Glu279, and Asn280 contribute to formation of the nucleotide binding pocket (Fig. 3b). The adenine ring is stabilized by Glu279 and Asn280 and the 2′-phosphate group of NADPH is stabilized by Gln219, Asn272, and Arg276 through hydrogen bonds. The Glu223 and Lys270 residues form hydrogen bonds with the ribose moiety (Fig. 3b). Two serine residues, Ser214 and Ser220, are involved in stabilizing pyrophosphate moiety and the nicotinamide ring of NADPH is stabilized by the residues Asp43, Tyr48, His110, Ser165, Asn166, Gln187, and Tyr213 through hydrogen bond networks (Fig. 3c).

**Table 2:** The kinetic analysis of *Ss*XR.

		K_m_ [mM]	*k*_cat_ [s^−1^]
0 mM NaCl	NADPH	0.00928 ± 0.00108	7.65 ± 0.23
	NADH	0.0187 ± 0.0026	2.68 ± 0.11
	xylose^NADPH^	32.37 ± 3.02	8.37 ± 0.26
	xylose^NADH^	39.61 ± 3.56	4.34 ± 0.14
150 mM NaCl	NADPH	0.0277 ± 0.0020	7.63 ± 0.19
	NADH	0.136 ± 0.017	2.39 ± 0.15
	xylose^NADPH^	39.40 ± 2.65	6.69 ± 0.16
	xylose^NADH^	59.72 ± 9.76	3.02 ± 0.20

Interestingly, when we superimposed the *Ss*XR structure in the apo-form with that in complex with NADPH, we observed a large structural difference near the NADPH binding site between these two structures (Fig. 3d). In the NADPH-bound form, the α9 helix and α9-α10 connecting loop (Gly217-Ser232) were in the closed conformation without crystal contact, and the Gln219 and Glu223 residues form hydrogen bonds with the phosphate atom of the phosphoribose moiety (Fig. 3b,d,e). In contrast, the region moved away from the NADPH binding site by 11.1 Å and formed an open conformation (Fig. 3d,f). Based on these observations, we propose that *Ss*XR undergoes an open/closed conformational change upon binding of the NADPH cofactor.

### _D_-Xylose binding mode of *Ss*XR

**Figure 4:**
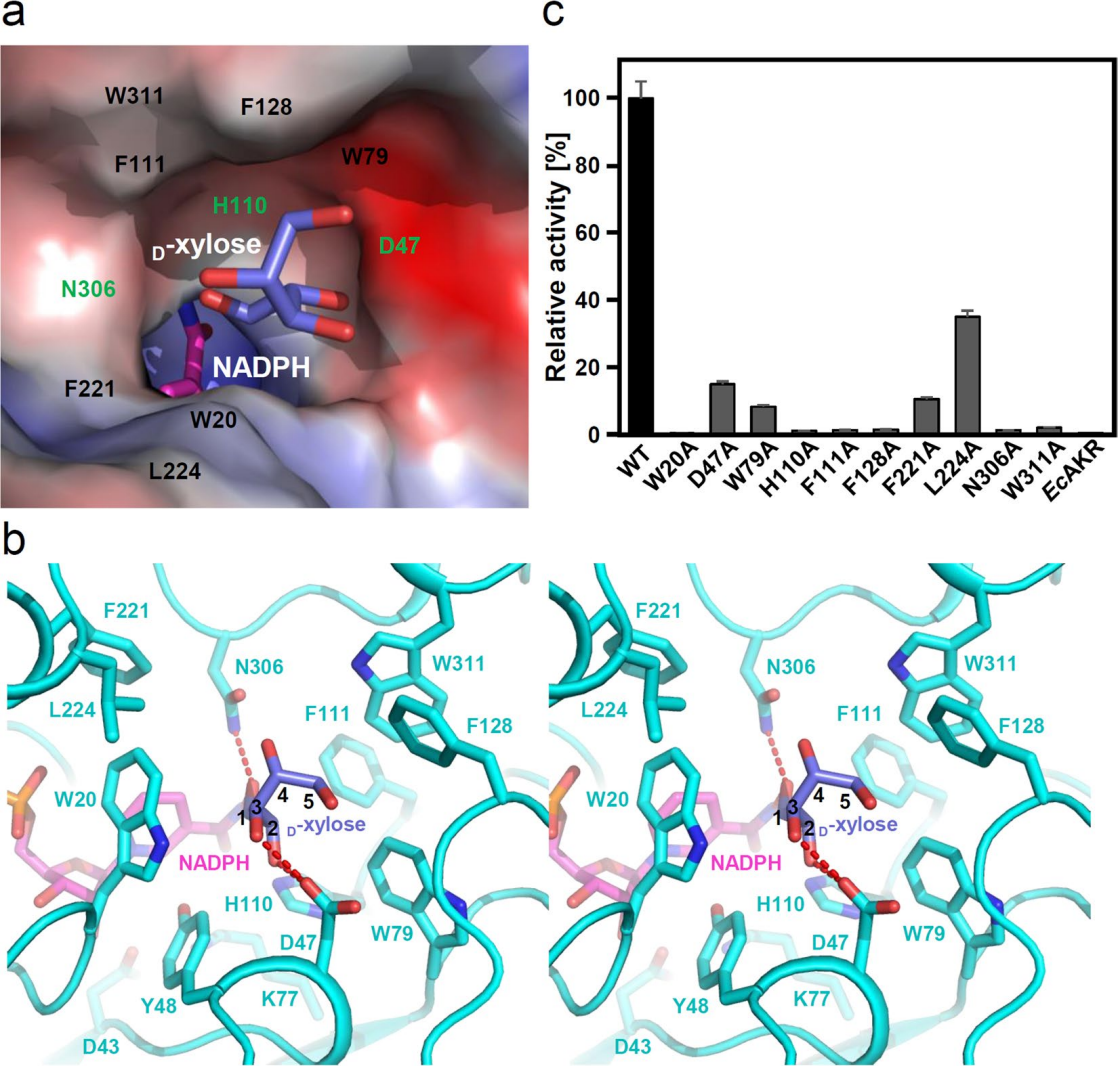
Substrate binding mode of *Ss*XR. (**a**) Electrostatic potential surface presentation of the _D_-xylose substrate binding mode of *Ss*XR. The *Ss*XR structure is shown as an electrostatic potential surface presentation using PYMOL software. The NADPH cofactor and _D_-xylose substrate presented by stick models with magenta and light-blue colors, respectively, and labeled. The sites of hydrophilic and hydrophobic residues involved in the substrate binding pocket formation were labelled with green and black colors, respectively. (**b**) Stereo-view of substrate binding mode of *Ss*XR. The *Ss*XR structure is shown as a cartoon diagram with cyan color. The residues involved in the substrate binding and catalysis are shown as stick models and labeled appropriately. The bound NADPH cofactor and modeled _D_-xylose substrate were shown as stick model, with magenta and light-blue colors, respectively, and labeled. Hydrogen bonds involved in the substrate binding are shown with red-colored dotted lines. 1 to 5 indicate the carbon number of the xylose substrate. (**c**) Site-directed mutagenesis of *Ss*XR. Residues involved in the _D_-xylose substrate binding are replaced by alanine residues. The relative activities of recombinant mutant proteins were measured and compared with that of wild-type *Ss*XR.

To elucidate the binding mode of the _D_-xylose substrate, we performed molecular docking simulations of *Ss*XR with the _D_-xylose substrate. Molecular docking simulations revealed that the _D_-xylose substrate fits well into the predicted substrate binding pocket (Fig. 4a). The _D_-xylose binding pocket is constituted by 10 residues: Trp20, Asp47, Trp79, His110, Phe111, Phe128, Phe221, Leu224, Asn306, and Trp311 (Fig. 4a,b). The Asp47 residue contributes to the stabilization of two hydroxyl groups (OH2 and OH3), and the aldehyde group of _D_-xylose is stabilized by Asn306 through hydrogen bonding (Fig. 4b). The residues involved in formation of the _D_-xylose binding pocket were confirmed by site-directed mutagenesis experiments (Fig. 4c). Most mutants, including W20A, D47A W79A, H110A, F111A, F128A, F221A, N306A, and W311A, exhibited almost complete loss of enzyme activity, indicating that these residues are crucial for _D_-xylose binding. However, the L224A mutant showed 35% XR activity compared to the wild type, indicating that the Leu224 residue does not influence substrate binding as much as other residues (Fig. 4b,c). Interestingly, the _D_-xylose binding pocket is somewhat hydrophobic and seven of 10 residues involved in formation of the _D_-xylose binding pocket are hydrophobic (Fig. 4a,b). Considering that the _D_-xylose substrate is highly hydrophilic with four hydroxyl groups and one aldehyde group, we can predict that the substrate binding pocket of *Ss*XR requires more hydrophilic residues. Thus, the hydrophobicity of the substrate binding site of *Ss*XR indicates that the binding affinity of the enzyme for _D_-xylose is low. We then performed kinetic analysis of the enzyme and the Km value for _D_-xylose was extremely high with a value of 39.4 mM (Table 2). Because the Km value of _D_-xylose was significantly higher than that of the NADPH cofactor (0.0277 mM) (Table 2), the bottleneck of the enzyme activity in *Ss*XR appears to be the binding affinity for _D_-xylose.

### Phylogenetic tree analysis and classification of XRs

**Figure 5:**
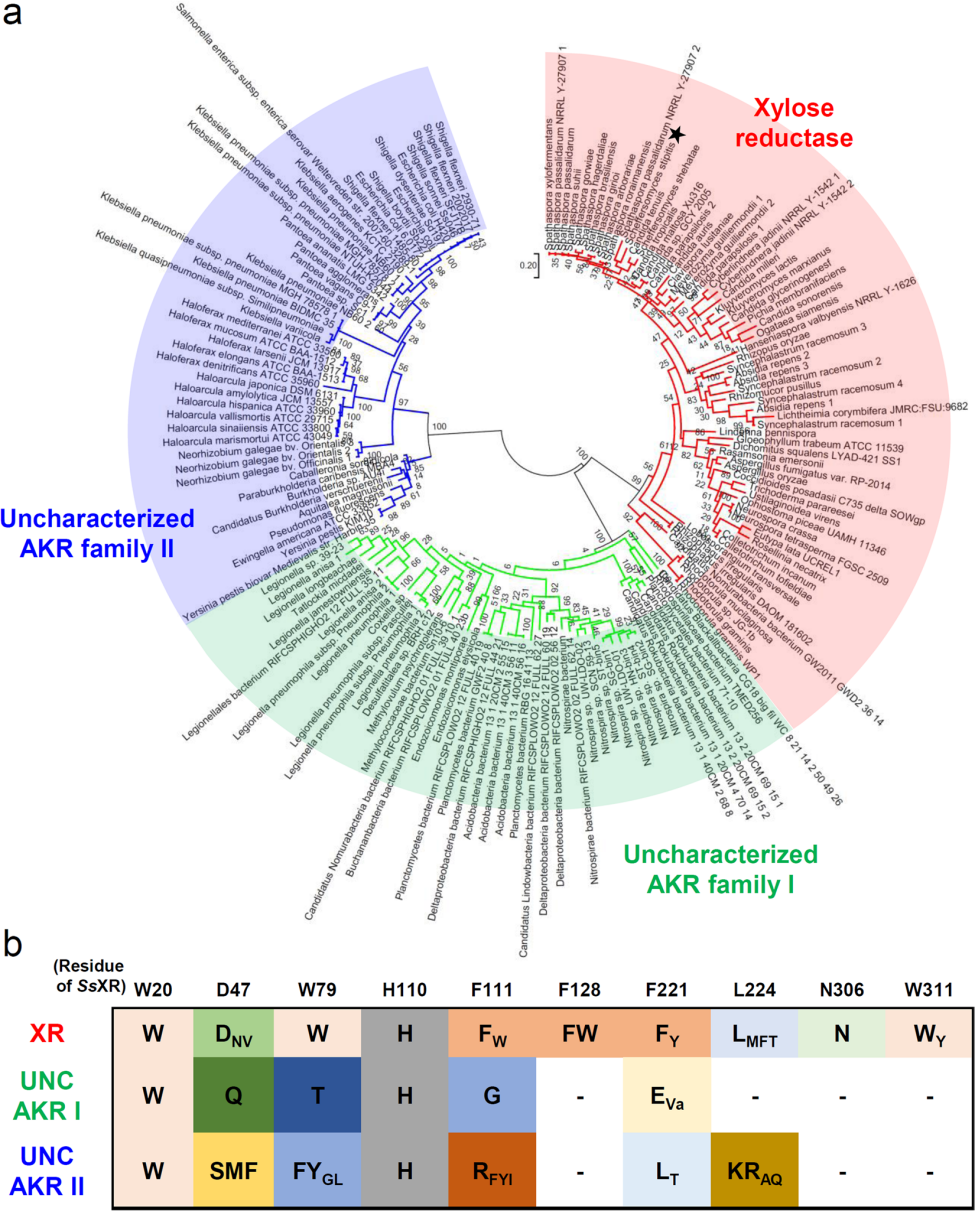
Phylogenetic analysis of XR proteins. (**a**) Unrooted maximum Likelihood tree of annotated XRs in NCBI. XRs, uncharacterized AKR family I and II are labeled as red, green, and blue colors, respectively, and labeled. *Ss*XR indicates by black colored star. The accession codes are listed at Supporting Table S1. (**b**) Amino acid sequence alignment of the residues involved in the substrate binding. Each amino acid is represented by single letter code, and ‘Va’ is an abbreviation for ‘Various’.

Because utilization of _D_-xylose by bio-fuel producing organisms, such as *S*. *cerevisiae* and *Y*. *lipolytica*, requires heterologous introduction of XR enzymes, selection of highly efficient XR is a key issue in high bio-fuel production^4^. In order to explore the XR candidates that can be introduced to microorganisms for xylose consumption, enzymes annotated as XR in NCBI and UNIPROT server were analyzed (Table. S1). There are 161 AKR enzymes (67 yeast/fungal, 83 bacterial, and 11 archaeal enzymes) annotated with XR, and these enzymes can be divided into three groups by phylogenetic tree analysis (Fig. 5a). Interestingly, only yeast and fungi derived enzymes belonged to the same group with *Ss*XR, and all other enzymes from bacteria and archaea belonged to the different group from *Ss*XR. Furthermore, amino acid sequence alignment also showed that 161 AKR enzymes were classified in the same way as the phylogenetic analysis, and only yeast/fungi derived enzymes have amino acid sequence similar to *Ss*XR (Fig. S2). In particular, when the residues involved in the substrate binding of *Ss*XR were compared each other, there are many gaps in bacterial/archaeal AKR enzymes (Fig. 5b). These observations indicate that AKR enzymes annotated with bacterial/archaeal XRs belonged to uncharacterized AKR families (UNC AKR I and II) and might have no XR function. In order to confirm that the enzymes belonging to UNK AKR families cannot use xylose as a substrate, we measured an XR activity using purified AKR from *E*. *coli*, an enzyme belonging to UNK ASK II (Accession codes of NCBI and UNIPROT are WP_001199831 and C3TM25, respectively), and the enzyme showed no enzyme activity against xylose (Fig. 4c). Consequently, we propose that the yeast/fungi derived enzymes, which belong to the same group with *Ss*XR, can be candidates for XR. In addition, when we compared to the amino acid residues involved in the formation of xylose binding pockets, only four of ten residues are conserved in the yeast/fungi derived XRs, and various amino acids are positioned in the xylose binding pocket (Figs 5b, S2). Therefore, structural and biochemical studies on other yeast/fungi derived XRs are required to select more efficient XRs and to increase biofuel productivity using lignocellulosic biomass.

We report the crystal structure of *Ss*XR in the apo form and in complex with the NADPH cofactor and provide structural insight into the open/closed conformational change upon cofactor binding. Molecular docking simulations of *Ss*XR for the _D_-xylose substrate and kinetic analysis of the protein revealed that why *Ss*XR shows low binding affinity for the substrate. Moreover, based on phylogenetic tree analysis and detailed amino acid sequence alignment, we propose that various yeast/fungi derived XRs can be candidate for more efficient XRs. These results may be useful for developing methods with much higher _D_-xylose utilization for lignocellulosic biomass in biofuel production.

## Material and Methods

### Cloning, expression and purification

The *Ss*XR coding gene was amplified through PCR from synthetic gene by Bioneer (Republic of Korea), and primers (forward: 5′-GCGC**CATATG**CCTTCTATTAAGTTGAACTCTGGTTAC-3′, reverse: 5′-GCGC**CTCGAG**TTAGACGAAGATAGGAATCTTGTCC-3′). The PCR product was digested by restriction enzymes (NdeI and XhoI), and sub-cloned into pProEX-HTa vector (Thermo Fischer Scientific), which contained a 6x -His tag with rTEV protease cleavage site at N-terminus. The pProEX-HTa:*Ss*XR was transformed into a *E*. *coli* BL21(DE3)-T1^R^ strain, and transformed *E*. *coli* was grown to an OD_600_ of 0.65 in LB medium containing 100 mg L^−1^ ampicillin at 310 K and *Ss*XR protein expression was induced by 0.5 mM isopropyl β-D-1-thiogalatopyranoside (IPTG). After 18 h at 293 K, the cell was harvested at 277 K. The cell pellet was resuspended in 40 mM Tris-HCl, pH 8.0 (buffer A) and disrupted by ultrasonication. The cell debris was removed by centrifugation at 13,000 g for 30 min, and the lysate was applied onto a Ni-NTA agarose column (Qiagen). After washing with 40 mM Tris-HCl, pH 8.0 and 25 mM Imidazole (buffer B), the bound proteins were eluted with 40 mM Tris-HCl, pH 8.0 and 300 mM Imidazole (buffer C). Finally, the trace amount of contaminants was removed by gel filtration experiment using HiPrep 26/60 Sephacryl S-300 HR column (GE Healthcare Life Sciences) equilibrate with buffer A. The eluted protein had about 75 kDa molecular weight, indicating a dimeric structure. The protein was concentrated to 75 mg mL^−1^, and kept at 193 K for further experiments.

### Crystallization of *Ss*XR

Crystallization of the *Ss*XR protein was initially performed with commercially available sparse-matrix screens, including PEG ion I and II, and Index, (Hampton Research), and Wizard Classic I and II (Rigaku Reagents), using the sitting-drop vapor diffusion method at 295 K. Each experiment consisted of mixing 1.0 μL protein solution (75 mg mL^−1^, buffer A) with 1.0 μL reservoir solution and then equilibrating against 50 uL reservoir solution. The apo-form of *Ss*XR crystals were observed from various crystallization conditions. After several steps for crystal improvement, crystals of the best quality appeared in 22% (w/v) polyethylene glycol 3350 and 0.1 M ammonium citrate tribasic, pH 7.0. For the crystal of the *Ss*XR in complex with NADPH cofactor, the *Ss*XR proteins was prepared in the same manner as that of apo form. The *Ss*XR protein was mixed with 20 mM NADPH cofactor, and incubated for 1 hr at 277 K. *Ss*XR crystals in complex with NADPH were crystallized in the condition of 20% (w/v) polyethylene glycol 3350 and 8% (v/v) Tacsimate, pH 7.0.

### Data collection and structure determination

The crystals of *Ss*XR in its apo form and in complex with NADPH were transferred to cryo-protectant solution containing 30% (v/v) glycerol with the crystallization buffer condition. The crystals were mounted in a 100 K nitrogen stream. Data of apo and NADPH complex were collected to a resolution of 1.95 and 2.0 Å, respectively, at 7 A beamline of the Pohang Accelerator Laboratory (PAL, Pohang, Republic of Korea), using a Quantum 270 CCD detector (ADSC, USA)^39^. All data were indexed, integrated, and scaled together using the HKL2000 software package^40^. The apo-form crystals of *Ss*XR belonged P2_1_2_1_2_1_ with unit cell parameters a = 69.248 Å, b = 87.151 Å, c = 122.62 Å, α = β = γ = 90°. Assuming two *Ss*XR molecules in asymmetric unit, the crystal volume per unit of protein mass was 2.58 Å^3^ Da^−1^, which means the solvent content was approximately 52.29%^41^. The crystals of *Ss*XR in complex with NADPH belonged to the space group P4_2_2_1_2, with unit cell parameters a = b = 97.654 Å, c = 160.12 Å, α = β = γ = 90°. Assuming two *Ss*XR molecules in asymmetric unit, the crystal volume per unit of protein mass was 2.73 Å^3^ Da^−1^, which means the solvent content was approximately 54.92%. The apo form structure of *Ss*XR was determined by molecular replacement with the CCP4 version of MOLREP^42^ using the structure of xylose reductase from *Candida tenuis* (*Ct*XR, PDB code 1Z9A) as a search model^43^. Model building was performed manually using WinCoot software ^44^, and refinement was performed with refmac5 ^45^. The structure of *Ss*XR in complex with NADPH was determined by molecular replacement using the crystal structure of the apo-form of *Ss*XR (PDB code 5Z6U). The data statistics are summarized in Table 1. The *Ss*XR structures of apo-form and in complex with NADPH were deposited in the protein data bank with PDB codes of 5Z6U and 5Z6T, respectively.

### Size-exclusion chromatographic analysis

To investigate the oligomeric state of *Ss*XR, analytical size-exclusion chromatography was performed using a Superdex increase 200 10/300 GL column (GE Healthcare Life Sciences) equilibrated with 40 mM Tris-HCl, pH 8.0 and various NaCl concentrations, such as 0, 50, 100, and 150 mM. Protein sample of 0.5 mL with concentration of 1 mg mL^−1^ was analyzed. In order to calculate the molecular weight of eluted *Ss*XR sample, the calibration curve was drawn using standard sample of ferritin (440 kDa), conalbumin (75 kDa), carbonic anhydrate (29 kDa), and ribonuclease A (13.7 kDa) (GE Healthcare Life Sciences).

### Kinetic analysis

For the kinetic analysis, the *Ss*XR protein was purified in the same manner as the protein for crystallization. The activities of *Ss*XR were determined by measuring the decrease of absorbance at 340 nm (extinction coefficient of 6.22 × 10^3^ M^−1^ cm^−1^). For the kinetic analysis of *Ss*XR on NADPH and NADH cofactor, enzyme activity was measured with a reaction mixture of 0.5 ml total volume at 303 K. The reaction mixture contained 100 mM Tris-HCl, pH 8.0, 100 mM _D_-xylose, and various concentration of NADPH/NADH cofactor from 1 to 200 μM. The reactions were initiated by the addition of enzyme to a final concentration of 0.5 and 2 μM for the analysis of NADPH and NADH, respectively. For the kinetic analysis of *Ss*XR on _D_-xylose substrate, enzyme reactions were performed with a reaction mixture of 0.5 ml total volume at 303 K. The reaction mixture contained 100 mM Tris-HCl, pH 8.0, 0.2 mM NADPH, and various concentrations of _D_-xylose substrate from 1 to 200 mM. The reaction was initiated by the addition of enzyme to a final concentration of 2 μM. The *Ss*XR activity measurement was performed in duplicate reaction.

### Molecular docking simulation

Molecular docking simulations of _D_-xylose to *Ss*XR structure was performed using AutoDock Vina software^46^. *Ss*XR structure of PDB code 5Z6T, in complex with NADPH, was used and the _D_-xylose ligand was prepared using the MarvinScketch software. The *pdbqt* files were generated by AutoDock Vina manual. Side-chain of Asp47, Tyr48, and Asn306 were treated as flexible residues, and the grid size for _D_-xylose was x = 18, y = 36, z = 40, and grid center was designated at x = 17.169, y = 21.352, z = 33.799. The final conformations produced in this simulation were confirmed using PyMOL software^47^. The calculated free energy of binding (ΔG_bind_) of the final pose was −4.5 kcal/mol.

### Site-directed mutagenesis and activity assay

Site-specific mutations were created with the QuikChange kit (Stratagene), and sequencing was performed to confirm correct incorporation of the mutations. The mutant proteins were purified in the same manner as the wild type. The activities of *Ss*XR were determined by measuring the decrease of absorbance at 340 nm (extinction coefficient of 6.22 × 10^3^ M^−1^ cm^−1^). Enzyme reactions for the relative activity of *Ss*XR were performed with a reaction sample of 0.5 ml total volume at 303 K. The reaction sample contained 100 mM Tris-HCl, pH 8.0, 100 mM _D_-xylose, and 200 μM NADPH. The reactions were initiated by the addition of enzyme to a final concentration of 0.5 μM. The *Ss*XR activity assay was performed in duplicate reaction.

### Phylogenetic tree analysis of reported XRs

Annotated XR enzymes are searched by protein search in National Center for Biotechnology Information (NCBI) and UNIPROT server. Multiple sequence alignment was performed using Clustal Omega^48^. The evolutionary history was inferred by using the Maximum Likelihood method based on the JTT matrix-based model^49^. The tree with the highest log likelihood (−19893.7669) is shown. Initial tree(s) for the heuristic search were obtained automatically by applying Neighbor-Join and BioNJ algorithms to a matrix of pairwise distances estimated using a JTT model, and then selecting the topology with superior log likelihood value. The tree is drawn to scale, with branch lengths measured in the number of substitutions per site. The analysis involved 161 amino acid sequences. All positions containing gaps and missing data were eliminated. There were a total of 133 positions in the final dataset. Evolutionary analyses were conducted in MEGA7^50^.

## Electronic supplementary material

Supplementary Information
