# Supplementary material for: Structural insight into D-xylose utilization by xylose reductase from Scheffersomyces stipitis
Source: Sci Rep. 2018 Nov 28;8:17442. doi: 10.1038/s41598-018-35703-x (PMC6261992; doi:10.1038/s41598-018-35703-x)
Supplement: Supplementary file 1 — Supplementary Information [file 41598_2018_35703_MOESM1_ESM.pdf]

Supplementary Information

Structueral insight into $_D$-xylose utilization by xylose reductase from *Scheffersomyces stipitis*

Hyeoncheol Francis Son[1,2], Sun-Mi Lee[3]* and Kyung-Jin Kim[1,2]*

[1]*School of Life Sciences, KNU Creative BioResearch Group, Kyungpook National University, Daegu 41566, Republic of Korea*

[2]*KNU Institute for Microorganisms, Kyungpook National University, Daegu 41566, Republic of Korea*

[3]*Clean Energy Research Center, Korea Institute of Science and Technology (KIST), Seoul 02792, Republic of Korea*

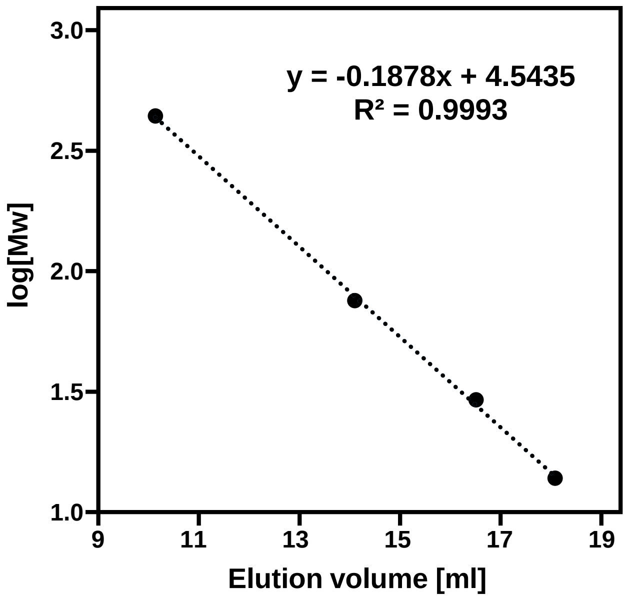

Standard sample	Mw (kDa)	Elution volume	log[Mw]
Ferritin	440	10.14	2.643
Conalbumin	75	14.11	1.875
Carbonic anhydrase	29	16.52	1.462
Ribonuclease A	13.7	18.09	1.137

	Elution volume (ml)	Calculated Mw (kDa)
0mM	13.85	87.59
50mM	15.10	51.02
100mM	15.53	42.36
150mM	15.77	38.19

Figure S1. Size exclusion chromatography.

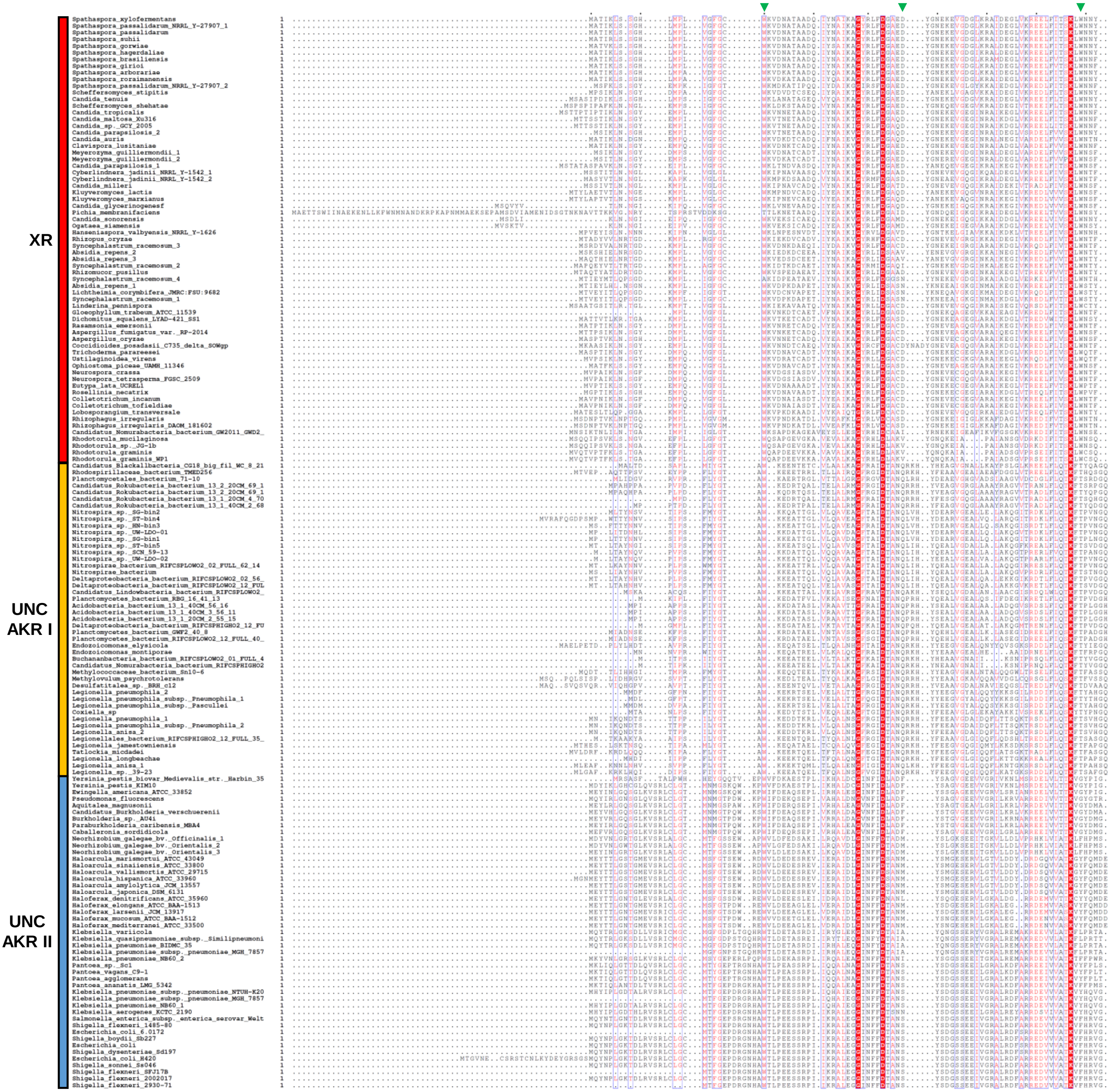

Figure S2. Amino acid sequence alignment of annotated XRs. Amino acid sequence alignment of xylose reductase (XR), uncharacterized aldo-keto reductase I (UNC AKR I), and uncharacterized aldo-keto reductase II (UNC AKR II) are distinguished by red, yellow, and blue colored bars, respectively. The residues involved in the formation of $_D$-xylose substrate binding pocket are indicated by green colored triangles.

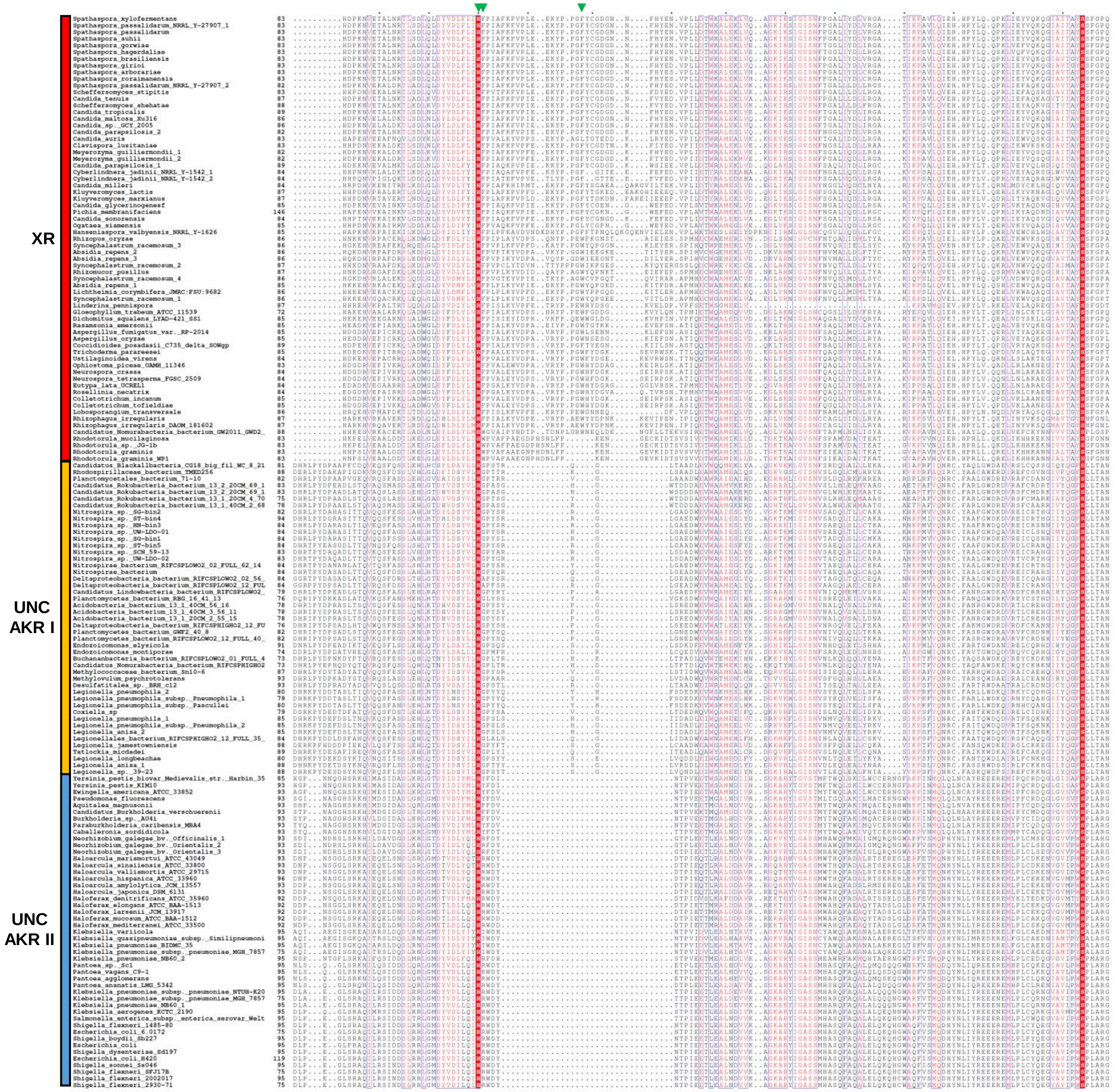

Figure S2. Amino acid sequence alignment of annotated XRs. (Continued)

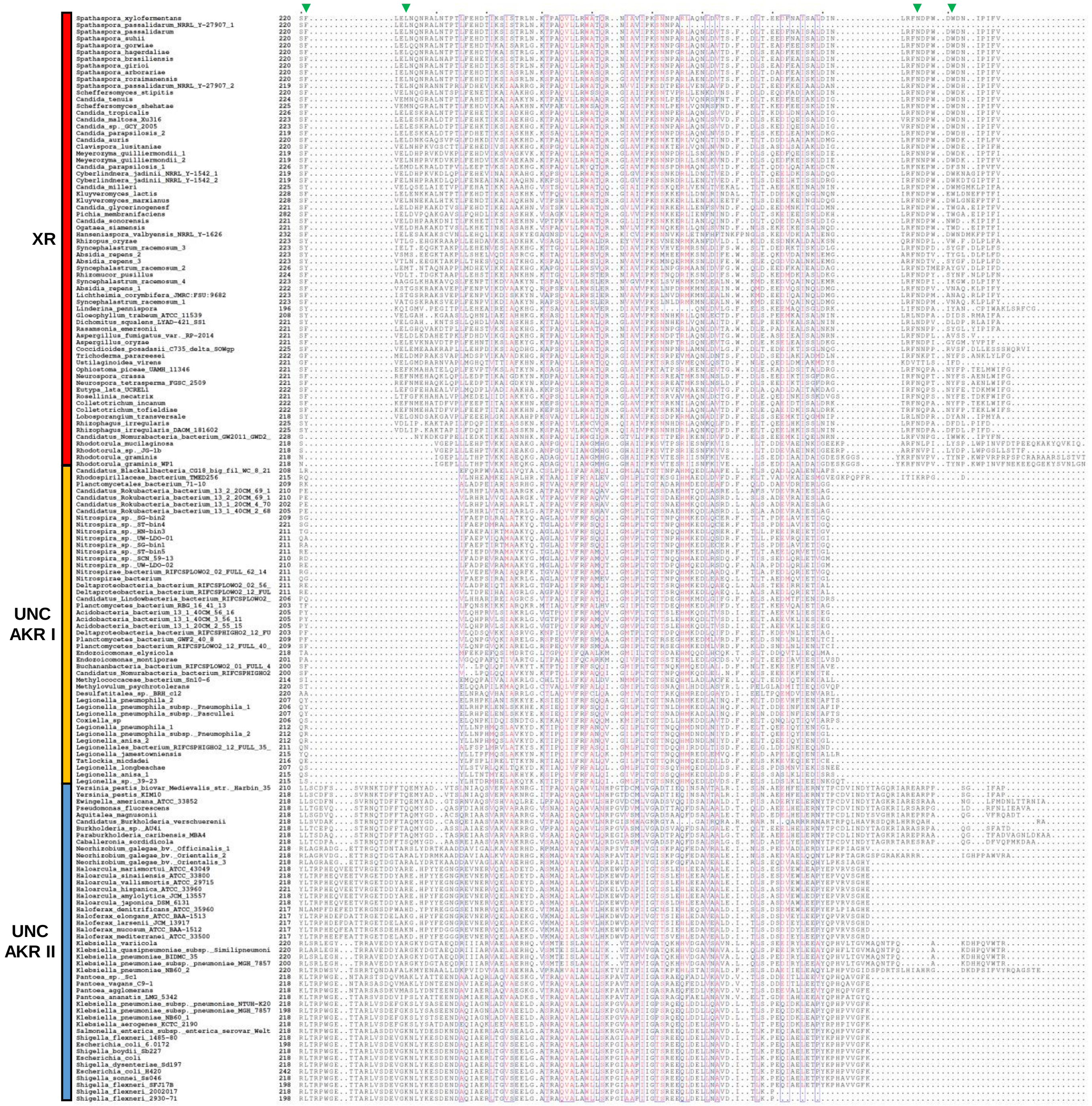

Figure S2. Amino acid sequence alignment of annotated XRs. (Continued)

Type	NCBI Accession Code	Uniprot Accession Code	Amino acids	Assignment
Xylose reductase (yeast/fungi)	**ORZ16479**	**A0A1X2IHA7**	321	Absidia_repens_1
	**ORZ15183**	**A0A1X2IEP5**	321	Absidia_repens_2
	**ORZ05368**	**A0A1X2HYK4**	321	Absidia_repens_3
	**KEY77854**	**A0A0J5PPW2**	315	Aspergillus_fumigatus_var._RP-2014
	**XP_001819987**	**Q2UKD0**	319	Aspergillus_oryzae
	**XP_018169903**	**A0A0L0P1P4**	318	Candida_auris
	**XP_020546229**	**A0A0S2E7S4**	320	Candida_glycerinogenesf
	**EMG46573**	**M3J3U0**	321	Candida_maltosa_Xu316
	**AGI78928**	**M9RYL7**	326	Candida_milleri
	**Q6Y0Z3**	**Q6Y0Z3**	324	Candida_parapsilosis_1
	**ABK32844**	**A0MSQ7**	317	Candida_parapsilosis_2
	**CCG55375**	**A0A0A8K9D1**	319	Candida_sonorensis
	**AAW34373**	**Q5I599**	321	Candida_sp._GCY_2005
	**O74237**	**O74237**	322	Candida_tenuis
	**XP_002546515**	**P87039**	324	Candida_tropicalis
	**XP_002619976**	**C4XYW2**	318	Clavispora_lusitaniae
	**XP_003071426**	**C5P0S3**	330	Coccidioides_posadasii_C735_delta_SOWgp
	**OHW92893**	**A0A1S1VKI6**	323	Colletotrichum_incanum
	**KZL67210**	**A0A166PVE3**	323	Colletotrichum_tofieldiae
	**XP_020069388**	**A0A1E4RYI9**	319	Cyberlindnera_jadinii_NRRL_Y-1542_1
	**XP_020071462**	**A0A1E4S4P9**	319	Cyberlindnera_jadinii_NRRL_Y-1542_2
	**KKY31113**	**A0A0G2H7D9**	355	Diaporthe_ampelina
	**XP_007360356**	***N/A***	318	Dichomitus_squalens_LYAD-421_SS1
	**XP_007794348**	**M7SQ33**	323	Eutypa_lata_UCREL1
	**XP_007860843**	**S7QLW8**	305	Gloeophyllum_trabeum_ATCC_11539
	**OBA26260**	**A0A1B7TC08**	336	Hanseniaspora_valbyensis_NRRL_Y-1626
	**XP_454929**	**P49378**	329	Kluyveromyces_lactis
	**AKN04691**	**A0A0H3Y344**	329	Kluyveromyces_marxianus
	**CDH61395**	**A0A068RJG4**	322	Lichtheimia_corymbifera_JMRC:FSU:9682
	**ORX68024**	**A0A1Y1W4D3**	293	Linderina_pennispora

Table S1. List of XRs annotated in NCBI and UNIPROT server used for phylogenetic tree analysis.

Type	NCBI Accession Code	Uniprot Accession Code	Amino acids	Assignment
Xylose reductase (yeast/fungi)	**XP_021883478**	**A0A1Y2GW55**	317	Lobosporangium_transversal
	**AAD09330**	***N/A***	317	Meyerozyma_guilliermondii_1
	**ABB87187**	***N/A***	317	Meyerozyma_guilliermondii_2
	**XP_963931**	**Q7SD67**	322	Neurospora_crassa
	**XP_009856077**	**G4U5N7**	322	Neurospora_tetrasperma_FGSC_2509
	**ACN78427**	**C0LSY9**	319	Ogataea_siamensis
	**EPE05500**	**S3CXC2**	322	Ophiostoma_piceae_UAMH_11346
	**GAV28788**	**A0A1Q2YGY6**	381	Pichia_membranifaciens
	**ACR78268**	***N/A***	320	Rasamsonia_emersonii
	**BAM28880**	**I7HD84**	322	Rhizomucor_pusillus
	**PKY54380**	***N/A***	321	Rhizophagus_irregularis
	**AER12712**	**A0A015K788**	321	Rhizophagus_irregularis_DAOM_181602
	**AHG97682**	**W5RZ21**	320	Rhizopus_oryzae
	**ADF57214**	**D6R257**	329	Rhodotorula_graminis
	**XP_018268405**	**A0A0P9ESV5**	329	Rhodotorula_graminis_WP1
	**ALO17776**	**A0A0S2I5X5**	324	Rhodotorula_mucilaginosa
	**KWU42507**	**A0A109FDK9**	313	Rhodotorula_sp._JG-1b
	**XP_016271038**	**M7X8C7**	326	Rhodotorula_toruloides_NP11
	**Q9P430**	**Q9P430**	323	Scheffersomyces_shehatae
	**XP_001385181**	**P31867**	318	Scheffersomyces_stipitis
	**ALP00838**	**A0A0S2PJH8**	318	Spathaspora_arborariae
	**ALP00839**	**A0A0S2PJR9**	318	Spathaspora_brasiliensis
	**ANG59288**	**A0A173DUJ7**	318	Spathaspora_girioi
	**ANG59287**	**A0A173DUJ9**	318	Spathaspora_gorwiae
	**ANG59286**	**A0A173DUJ2**	318	Spathaspora_hagerdaliae
	**AGO89463**	**S4WCW2**	318	Spathaspora_passalidarum
	**XP_007375536**	**S4WCW2**	318	Spathaspora_passalidarum_NRRL_Y-27907_1
	**XP_007375534**	**G3APT8**	317	Spathaspora_passalidarum_NRRL_Y-27907_2
	**ALP00842**	**A0A0S2PJI3**	318	Spathaspora_roraimanensis
	**ALP00841**	**A0A0S2PJU9**	318	Spathaspora_suhii

Table S1. List of XRs annotated in NCBI and UNIPROT server used for phylogenetic tree analysis. (Continued)

Type	NCBI Accession Code	Uniprot Accession Code	Amino acids	Assignment (Organism)
**Type I Yeast/Fungi**	**ALP00840**	**A0A0S2PJP5**	318	Spathaspora_xylofermentans
	**ORZ01166**	**A0A1X2HQJ7**	322	Syncephalastrum_racemosum_1
	**ORY98712**	**A0A1X2HI69**	326	Syncephalastrum_racemosum_2
	**ORY93071**	**A0A1X2H3Z4**	321	Syncephalastrum_racemosum_3
	**ORY89922**	**A0A1X2GZP1**	322	Syncephalastrum_racemosum_4
	**OTA00903**	***N/A***	323	Trichoderma_parareesei
	**KDB16557**	**A0A063C520**	313	Ustilaginoidea_virens
**Uncharacterized AKR I (Bacteria)**	**OLE88765**	**A0A1Q8B9S6**	273	Acidobacteria_bacterium_13_1_20CM_2_55_15
	**OLD22713**	**A0A1Q7NCR5**	273	Acidobacteria_bacterium_13_1_40CM_3_56_11
	**OLC28161**	**A0A1Q7FMG7**	273	Acidobacteria_bacterium_13_1_40CM_56_16
	**OGY53552**	**A0A1G1YMG4**	266	Buchananbacteria_bacterium_RIFCSPLOWO2_01_FULL_40_23b
	**PIQ25289**	***N/A***	277	Candidatus_Blackallbacteria_CG18_big_fil_WC_8_21_14_2_50_49_26
	**OGH59037**	**A0A1F6LI14**	275	Candidatus_Lindowbacteria_bacterium_RIFCSPLOWO2_12_FULL_62_27
	**OGI66195**	**A0A1F6V9B1**	265	Candidatus_Nomurabacteria_bacterium_RIFCSPHIGHO2_01_FULL_39_10_1
	**OLD74683**	**A0A1Q7SEJ0**	270	Candidatus_Rokubacteria_bacterium_13_1_20CM_4_70_14
	**OLD39511**	**A0A1Q7PJB8**	273	Candidatus_Rokubacteria_bacterium_13_1_40CM_2_68_8
	**OLB03939**	**A0A1Q6VST3**	278	Candidatus_Rokubacteria_bacterium_13_2_20CM_69_15_1
	**OLA96595**	**A0A1Q6V6W4**	278	Candidatus_Rokubacteria_bacterium_13_2_20CM_69_15_2
	**PHQ81372**	***N/A***	276	Coxiella_sp
	**OGQ28647**	**A0A1F9GLC6**	271	Deltaproteobacteria_bacterium_RIFCSPHIGHO2_12_FULL_44_21
	**OGQ55333**	**A0A1F9IGZ4**	279	Deltaproteobacteria_bacterium_RIFCSPLOWO2_02_56_12
	**OGQ79615**	**A0A1F9KG48**	279	Deltaproteobacteria_bacterium_RIFCSPLOWO2_12_FULL_60_19
	**KJS28286**	**A0A0F2QRA9**	290	Desulfatitalea_sp._BRH_c12
	**WP_020585011**	**A0A081KAB6**	286	Endozoicomonas_elysicola
	**WP_034874283**	**A0A081N7L2**	271	Endozoicomonas_montiporae
	**WP_019232360**	**A0A0W0RM18**	284	Legionella_anisa_1
	**WP_010947778**	**Q5ZTU6**	280	Legionella_anisa_2
	**WP_058450499**	**A0A0W0UKU2**	285	Legionella_jamestowniensis
	**WP_003631635**	**D3HSQ9**	277	Legionella_longbeachae
	**WP_010947778**	**A0A133WXA6**	280	Legionella_pneumophila_1

Table S1. List of XRs annotated in NCBI and UNIPROT server used for phylogenetic tree analysis. (Continued)

Type	NCBI Accession Code	Uniprot Accession Code	Amino acids	Assignment (Organism)
**Uncharacterized AKR I (Bacteria)**	**KXB27056**	**A0A128LUG5**	277	Legionella_pneumophila_2
	**AMP88661**	**A0A140J1N0**	277	Legionella_pneumophila_subsp._Pascullei
	**WP_010946265**	**Q5ZTU6**	280	Legionella_pneumophila_subsp._Pneumophila_1
	**WP_010947778**	**A0A0W0S2B0**	280	Legionella_pneumophila_subsp._Pneumophila_2
	**OJW12115**	**A0A1Q3U8B5**	286	Legionella_sp._39-23
	**OGV28470**	**A0A1G0X481**	279	Legionellales_bacterium_RIFCSPHIGHO2_12_FULL_35_11
	**WP_045780034**	**A0A0F3IG30**	283	Methylococcaceae_bacterium_Sn10-6
	**WP_088617704**	**A0A1Z4BU30**	291	Methylovulum_psychrotolerans
	**OQW49354**	**A0A1W9HQ37**	279	Nitrospira_sp._HN-bin3
	**ODT46272**	**A0A1E4DQ68**	278	Nitrospira_sp._SCN_59-13
	**OQW37605**	**A0A1W9GQZ4**	279	Nitrospira_sp._SG-bin1
	**OQW37758**	**A0A1W9GRJ5**	277	Nitrospira_sp._SG-bin2
	**OQW56439**	**A0A1W9I9R3**	289	Nitrospira_sp._ST-bin4
	**OQW65277**	**A0A1W9J035**	279	Nitrospira_sp._ST-bin5
	**OYT18851**	**A0A256WSI4**	279	Nitrospira_sp._UW-LDO-01
	**OYT22449**	***N/A***	278	Nitrospira_sp._UW-LDO-02
	**PHX90169**	***N/A***	279	Nitrospirae_bacterium
	**OGW68169**	**A0A1G1IJE0**	279	Nitrospirae_bacterium_RIFCSPLOWO2_02_FULL_62_14
	**OJW27060**	**A0A1U7GS21**	277	Planctomycetales_bacterium_71-10
	**OHB48001**	**A0A1G2XPW6**	278	Planctomycetes_bacterium_GWF2_40_8
	**OHB72497**	**A0A1G2ZP69**	271	Planctomycetes_bacterium_RBG_16_41_13
	**OHC01583**	**A0A1G3C287**	278	Planctomycetes_bacterium_RIFCSPLOWO2_12_FULL_40_19
	**OUX28340**	**A0A1Z9SHU8**	299	Rhodospirillaceae_bacterium_TMED256
	**WP_045098019**	**A0A098GBS8**	284	Tatlockia_micdadei
**Uncharacterized AKR II (Bacteria)**	**WP_089083668**	**A0A224W1U6**	342	Aquitalea_magnusonii
	**WP_021160515**	**U2G1M1**	340	Burkholderia_sp._AU4i
	**WP_089162865**	**A0A226WXR7**	345	Caballeronia_sordidicola
	**KND57631**	**A0A0L0M5T7**	328	Candidatus_Burkholderia_verschuerenii
	**WP_001199831**	**C3TM25**	324	Escherichia_coli
	**EKK36593**	***N/A***	304	Escherichia_coli_6.0172

Table S1. List of XRs annotated in NCBI and UNIPROT server used for phylogenetic tree analysis. (Continued)

Type	NCBI Accession Code	Uniprot Accession Code	Amino acids	Assignment (Organism)
Uncharacterized AKR II (Bacteria)	**OSL66097**	**A0A1X3L7U2**	328	Escherichia_coli_H420
	**WP_034794324**	**A0A085G4M8**	347	Ewingella_americana_ATCC_33852
	**WP_015704564**	**A0A0H3FX88**	324	Klebsiella_aerogenes_KCTC_2190
	**WP_023301506**	**A0A169IR52**	339	Klebsiella_pneumoniae_BIDMC_35
	**WP_004893898**	**A0A1Y5CAT2**	324	Klebsiella_pneumoniae_NB60_1
	**EWD02768**	***N/A***	355	Klebsiella_pneumoniae_NB60_2
	**ABR78709**	**A6TDN8**	319	Klebsiella_pneumoniae_subsp._pneumoniae_MGH_78578_1
	**ABR75823**	**A6T5F2**	304	Klebsiella_pneumoniae_subsp._pneumoniae_MGH_78578_2
	**BAH62023**	**J2DPA8**	324	Klebsiella_pneumoniae_subsp._pneumoniae_NTUH-K2044
	**WP_023319107**	**B5XUG5**	339	Klebsiella_quasipneumoniae_subsp._Similipneumoniae
	**KMI11412**	***N/A***	339	Klebsiella_variicola
	**WP_046627503**	**A0A0T7FIT9**	324	Neorhizobium_galegae_bv._Officinalis_1
	**CDZ67293**	**A0A0T7HN79**	344	Neorhizobium_galegae_bv._Orientalis_2
	**WP_046600646**	***N/A***	324	Neorhizobium_galegae_bv._Orientalis_3
	**WP_033756762**	**A0A059IGF0**	324	Pantoea_agglomerans
	**WP_014593069**	**A0A0H3KT92**	324	Pantoea_ananatis_LMG_5342
	**WP_009091739**	***N/A***	324	Pantoea_sp._Sc1
	**WP_013356979**	**E1SF77**	324	Pantoea_vagans_C9-1
	**WP_035999131**	**A0A0P0RHG3**	348	Paraburkholderia_caribensis_MBA4
	**WP_063029576**	**A0A143GGP0**	344	Pseudomonas_fluorescens
	**WP_001199865**	**Q8Z8X4**	324	Salmonella_enterica_subsp._enterica_serovar_Weltevreden_str._2007-60-3289-1
	**ABB65026**	**I6EU46**	324	Shigella_boydii_Sb227
	**WP_001199782**	**Q32JH7**	324	Shigella_dysenteriae_Sd197
	**EJZ68269**	**K0XKD8**	324	Shigella_flexneri_1485-80
	**OUZ64738**	**D2A8G9**	305	Shigella_flexneri_2002017
	**EGJ98707**	**A0A226K6M7**	285	Shigella_flexneri_2930-71
	**EGM63403**	***N/A***	304	Shigella_flexneri_SFJ17B
	**WP_001199830**	**Q3Z4Z0**	324	Shigella_sonnei_Ss046
	**ADV98279**	***N/A***	331	Yersinia_pestis_biovar_Medievalis_str._Harbin_35
	**AAM85296**	**Q7CJ36**	339	Yersinia_pestis_KIM10

Table S1. List of XRs annotated in NCBI and UNIPROT server used for phylogenetic tree analysis. (Continued)

Type	NCBI Accession Code	Uniprot Accession Code	Amino acids	Assignment (Organism)
**Uncharacterized AKR II (Archaea)**	**WP_008309812**	**M0KJZ2**	325	Haloarcula_amylolytica_JCM_13557
	**AEM56771**	**G0HXN9**	328	Haloarcula_hispanica_ATCC_33960
	**WP_004593159**	**M0L7S1**	325	Haloarcula_japonica_DSM_6131
	**WP_011223046**	**Q5V4P5**	325	Haloarcula_marismortui_ATCC_43049
	**WP_004962368**	**M0K080**	325	Haloarcula_sinaiiensis_ATCC_33800
	**WP_004515687**	**M0JQA4**	325	Haloarcula_vallismortis_ATCC_29715
	**WP_004968192**	**M0JFY3**	324	Haloferax_denitrificans_ATCC_35960
	**WP_008323859**	**M0HRV8**	324	Haloferax_elongans_ATCC_BAA-1513
	**WP_007539636**	**M0HGI9**	324	Haloferax_larsenii_JCM_13917
	**WP_004572431**	**I3R3Z7**	324	Haloferax_mediterranei_ATCC_33500
	**WP_008321083**	**M0I5U4**	324	Haloferax_mucosum_ATCC_BAA-1512

Table S1. List of XRs annotated in NCBI and UNIPROT server used for phylogenetic tree analysis. (Continued)
